# *JPhyloIO*: a Java library for event-based reading and writing of different phylogenetic file formats through a common interface

**DOI:** 10.1186/s12859-019-2982-3

**Published:** 2019-07-22

**Authors:** Ben C. Stöver, Sarah Wiechers, Kai F. Müller

**Affiliations:** Institute for Evolution and Biodiversity, WWU Münster, Hüfferstraße 1, 48149 Münster, Germany

**Keywords:** Phylogenetic metadata, Data reuse, Data annotation, *NeXML*, *PhyloXML*, *NEXUS*, Phylogenetic tree, Multiple sequence alignment

## Abstract

**Background:**

Today a variety of phylogenetic file formats exists, some of which are well-established but limited in their data model, while other more recently introduced ones offer advanced features for metadata representation. Although most currently available software only supports the classical formats with a limited metadata model, it would be desirable to have support for the more advanced formats. This is necessary for users to produce richly annotated data that can be efficiently reused and make underlying workflows easily reproducible. A programming library that abstracts over the data and metadata models of the different formats and allows supporting all of them in one step would significantly simplify the development of new and the extension of existing software to address the need for better metadata annotation.

**Results:**

We developed the Java library *JPhyloIO*, which allows event-based reading and writing of the most common alignment and tree/network formats. It allows full access to all features of the nine currently supported formats. By implementing a single *JPhyloIO*-based reader and writer, application developers can support all of these formats. Due to the event-based architecture, *JPhyloIO* can be combined with any application data structure, and is memory efficient for large datasets. *JPhyloIO* is distributed under *LGPL*. Detailed documentation and example applications (available on http://bioinfweb.info/JPhyloIO/) significantly lower the entry barrier for bioinformaticians who wish to benefit from *JPhyloIO*’s features in their own software.

**Conclusion:**

*JPhyloIO* enables simplified development of new and extension of existing applications that support various standard formats simultaneously. This has the potential to improve interoperability between phylogenetic software tools and at the same time motivate usage of more recent metadata-rich formats such as *NeXML* or *phyloXML*.

## Background

The amount of available data in organismic and biodiversity-related disciplines, such as phylogenetics, taxonomy or ecology [1, 2] as well as related fields of molecular biology, especially genomics or genome evolution has been growing and continues to grow at an accelerated rate. Among other factors, increasingly cheaper high-throughput sequencing technologies [3], data collected in the context of barcoding initiatives [4–6], the ongoing digitization of biological collections [7, 8], and large-scale data acquisition (e.g., related to monitoring biodiversity) in citizen-science [9–12] contribute to this increasing amount of primary data. On top of that, the availability of faster processing units allows for increasingly advanced downstream analyses and the parallel application of multiple alternative methods and parameter sets, which in turn leads to even more (derived) data, potentially multiplying the amount with value for reuse in subsequent studies.

While these developments open up new perspectives for studies and applications that make use of big data, the practical reusability of data continues to be an issue. Primary sequence data tend to be reused on a regular basis, but the accessibility of other derived data types like phylogenetic trees is still low [13]. Phylogenetic hypotheses, for example, are often provided as image files only. Although software exists to reconstruct phylogenetic trees from images [14–17], making trees easily reusable would require representing tree topologies and branch lengths in defined phylogenetic file formats [13, 18]. Public availability and searchability of scientific data are necessary to foster reuse [19, 20] and can be addressed by respective policies of funding agencies and scientific journals [21] together with cyberinfrastructure (e.g., [22–26]) for the long-term storage. Data annotation is an equally important requirement to easily and unambiguously identify and understand relevant data that fits the need for a concrete project [13, 27, 28]. Metadata annotation is necessary to, e.g., unambiguously link tree nodes to sequences in a multiple sequence alignment that was used to generate the tree, or to link tree nodes and sequences to taxonomic information, ideally also using a taxonomic ID (e.g., *NCBI Taxonomy* [29]) - or even better linking a sequence back to the individual specimen it was derived from [30]. Additionally, linking relevant external resources (e.g., voucher information, digitized specimens or sequencing raw data) and providing metadata that reliably identifies the methods that were used to generate data (e.g., the software and parameters used for a phylogenetic inference) would further improve reusability of data and reproducibility of studies. More extensive lists of information to be provided for better reusability and reproducibility can be found in [31, 32]. Storing the results of phylogenetic analyses using metadata-rich formats is an ideal basis to link all necessary metadata and resources.

The big data-driven development of databases to reuse phylogenetic information (e.g., [24–26]) or the application of deep learning approaches (e.g., [33–35]) would be significantly simplified if more data were annotated with more structured metadata and could be automatically collected from a wide variety of studies. Beyond that, making phylogenetic data more reusable is important for every discipline where data is analyzed in a phylogenetic context, evolutionary aspects are part of studies, or alignments by homology or phylogenetic trees are needed at some point during analysis.

Phylogenetic file formats to store taxon or OTU lists, character matrices, multiple sequence alignments or phylogenetic trees and networks, are an integral part of phylogenetic workflows and the basis for interoperability between the software tools and databases involved. Several formats exist, which can be grouped into two categories: (i) classical formats, such as *FASTA*, *PHYLIP* [36], *Newick* [37] or *NEXUS* [38] and (ii) more recently developed formats with significantly more advanced metadata models, such as *NeXML* [39] or *phyloXML* [40] (See also Table 1). Due to their ability to store rich metadata, the more advanced formats are much better suited to annotate data in a way that allows efficient reuse and clear reproducibility, as argued above. The metadata model of *NeXML*, for example*,* uses *RDF*-predicates from externally defined ontologies to link external resources, allowing maximal reusability of phylogenetic data (*RDF* = *Resource Description Framework*, a semantic web technology to formulate logical statements, [41, 42]). These modern formats are *XML*-based and therefore well-defined by *XML schema* definitions. *XML* libraries available for nearly all programming languages can be used to process them. In contrast, the classical formats are plain text-based and usually do not have a formal definition (e.g., a grammar), resulting in different dialects and incomplete reader implementations. This frequently leads to interoperability issues when exchanging data between different software, which is another downside of using the classical formats instead of the more recent ones. In practice though, the classical formats dominate and only a small number of researchers actually make use of these newer formats and annotation standards. One important reason for this is that the majority of widely used computer applications only support the classical formats.

**Table 1:** **Formats supported by JPhyloIO**

Format	Type	OTUs	MSAs	Trees	Networks	Simple Metadata^1^	Complex metadata^2^	Ontologies^3^	Read	Write
*FASTA*	Text		X						X	X
*PHYLIP*	Text		X						X	X
*Relaxed PHYLIP*	Text		X						X	X
*NEXUS*	Text	X	X	X		X			X	X
*Newick*	Text			X		X			X	X
*eNewick (Newick, NEXUS)*	Text			X	X	X			X	
*NeXML*	XML	X	X	X	X	X	X	X	X	X
*phyloXML*	XML			X	X	X	X	3	X	X
*MEGA*	Text		X						X	
*PDE*	XML		X			X			X	
*XTG*	XML			X		X			X	

A variety of file formats used in phylogenetics are supported. These can either be based on *XML*, or define custom types of structured text which is indicated in the second column. The central columns show whether a format supports taxon/OTU lists, multiple sequence alignments, phylogenetic trees or networks and what type of metadata can be attached to at least some of these elements or their subelements. As shown in the two rightmost columns, *JPhyloIO* can read and write many of the common formats, while formats specific to single applications can only be read

^1^Attaching simple numeric or textual values to data elements

^2^Attaching complex metadata elements that may be represented as *XML* structures

^3^Simple annotations can be linked using *CURIE*-like identifiers and custom *XML* tags can be added to all elements but no explicit reference to external ontologies is currently supported in *phyloXML*

To help with the transition of the community to providing richly annotated data, software is required that simplifies such annotation and supports the modern formats. This software should allow importing from and exporting to the classical formats to provide downwards compatibility and be interoperable with applications that do not (yet) support metadata-rich formats. The development of such software can be costly and complicated since different readers and writers for all formats need to be implemented and the representation of the applications’ data in the different formats must be developed, which usually requires detailed knowledge on all formats. Since the necessary resources would have to be subtracted from working on the core functionality of the application, this is usually omitted. To foster the development of new and the extension of existing software to support the necessary variety of formats, we created the *Java* programming library *JPhyloIO*. It allows access to phylogenetic data through one common interface that fully models the data and metadata concepts of all mentioned formats. This enables *Java* application developers to support both modern metadata-rich formats (to produce easily reusable data) and classical formats (for larger interoperability) by implementing only one *JPhyloIO*-based reader and writer. *JPhyloIO* models data and metadata in all formats (as far as they allow this) without the need for the developer to explicitly deal with this problem, which we hope will help encourage the use of modern metadata-rich formats.

### Implementation

**Fig. 1:**
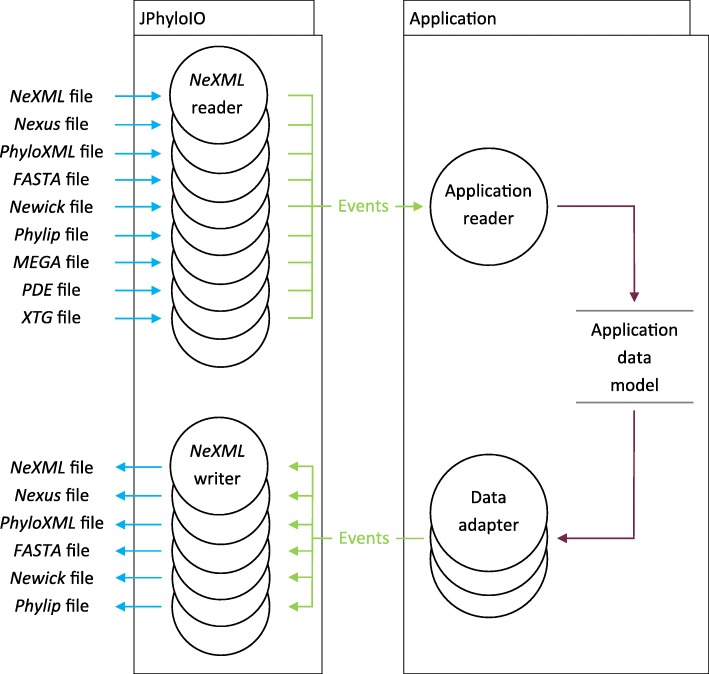
Data flow diagram showing how data is read into and written from an application data model. *JPhyloIO* contains a reader for each format that translates the contents of a file to a sequence of events that are then processed by the custom reader of an application. This reader has knowledge of the specific application data model and stores relevant information there. The writers available in *JPhyloIO* access the contents of that model using data adapters provided by the application that allow random access to the application’s data model. (For supported formats specific for a single application, only readers are provided)

**Fig. 2:**
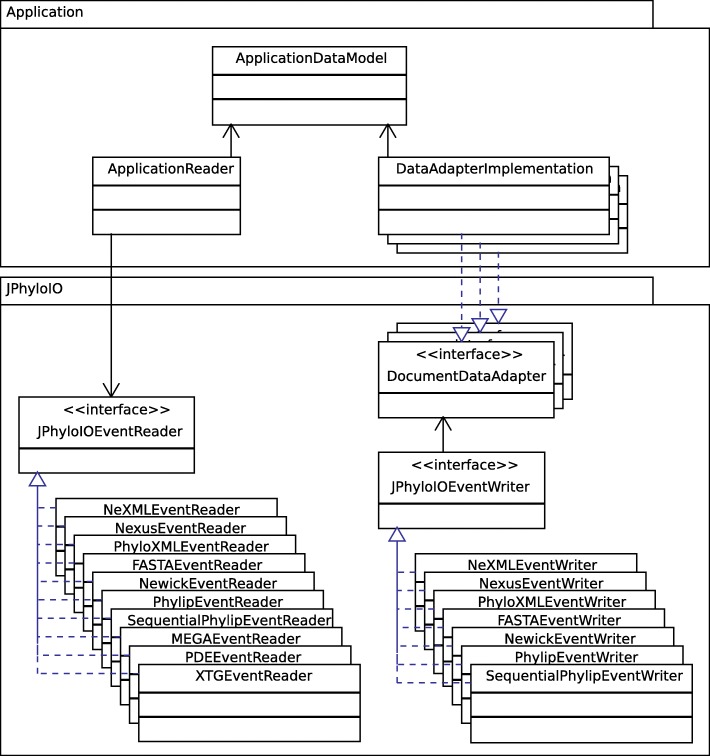
UML class diagram showing the relation between *JPhyloIO* and an application based on it. All readers and writers implement a common interface to be easily exchangeable in the application. Event readers produce a sequence of events (see Fig. 1) processed by an application reader class that acts as an adapter between *JPhyloIO* and the application data model. Conversely, a set of data adapter implementations of the application allows the *JPhyloIO* writers to access the data. Writing needs a slightly more complex architecture than reading, because writers need to access that data in different orders depending on the target format. To achieve this, a set of data adapters (see Fig. 5 For details) is necessary, each providing a subsequence of the whole event stream modelling a document

*JPhyloIO* offers a general way for reading and writing various phylogenetic file formats, without imposing any constraints on the data model of applications using the library. (See Fig. 1 and Fig. 2.) An event-based architecture (similar to iterator-based *StAX* for *XML* parsing that is common in *Java*) was chosen over a model-based approach (that would define its own data model classes), because representing phylogenetic data as a sequence of event objects allows compatibility with all application data structures and memory efficient processing. The *JPhyloIO* readers and writers for different formats all implement a common interface (using the strategy pattern [43]) and instances of them are created using a factory implementation [43] that can guess the format from a file or input stream.

### Event streams for reading documents

**Fig. 3:**
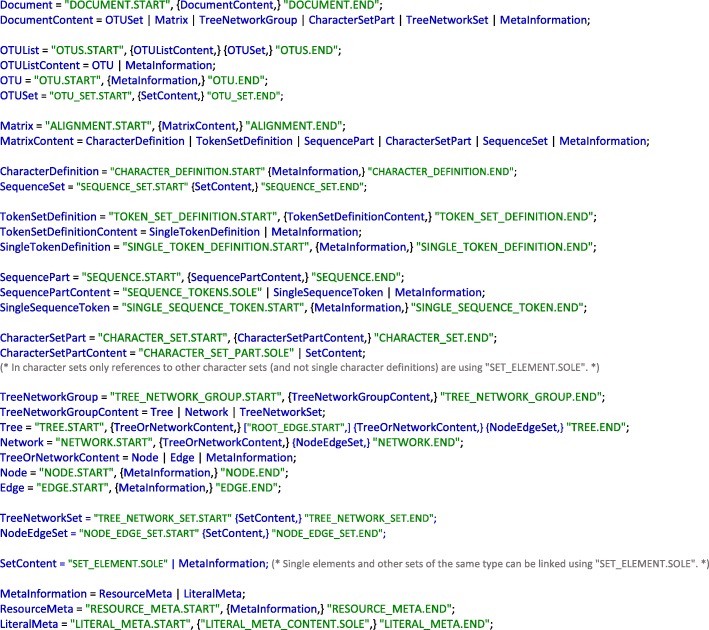
Grammar describing the event sequence generated by *JPhyloIO* readers. These readers translate the hierarchical data structure of a phylogenetic file (e.g., a NeXML file consisting of an alignment and a tree, which again consist of sequences or nodes and edges, and so forth) into a sequence of events as defined by this grammar in extended Backus-Naur form (EBNF). The terminal symbols (in green) represent the types of events, each of which either has a single SOLE or a START and END version, depending on whether additional data can be nested or not

**Fig. 4:**
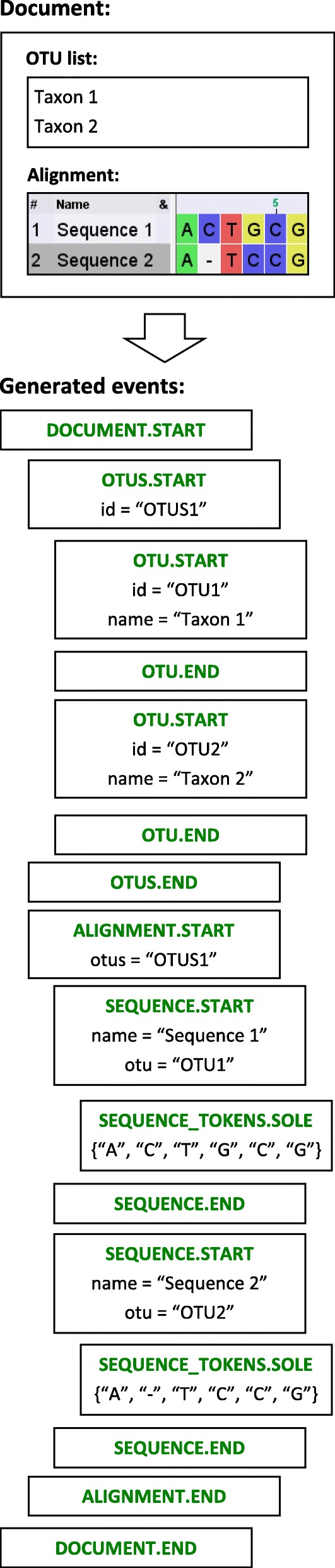
Example document with its respective event sequence. The document contains an OTU list and an alignment, which references this list. The event sequence is generated by a *JPhyloIO* reader (see also Fig. 2), where each box represents one event. Each has an ID in order to be referenced by subsequent events, as exemplarily shown by the OTU list and OTU start events, which are referenced by the related alignment and sequence start events

*JPhyloIO*‘s reader classes translate the hierarchical data structure of a document with phylogenetic data into a linear sequence of event objects, which is formally described by the grammar in Fig. 3. Events representing data elements that consist of smaller parts (e.g., an alignment that consists of sequences) are modelled as a pair of a start and an end event. The subsequence between such two consists of events that model the content of the data element at hand. By applying this recursively, the hierarchical structure of a document can be serialized to a linear event stream, as shown in the example in Fig. 4. Applications using *JPhyloIO* need to implement a reader for processing the encountered events and storing relevant information in their data structure (Fig. 1, Fig. 2). This can be done by iterating over the event stream using (*StaX*-like) pull parsing, which allows the application to actively request events one by one and therefore keep the control flow. Classes for (*SAX*-like) push parsing are additionally available, if an inversion of control is beneficial, e.g., if multiple event listeners need to be present on the application side. One such application reader implementation allows access to all supported formats, even when additional formats are added in future releases of *JPhyloIO*, without the need for any format-specific logic.

All created event objects have a string ID, which is unique inside a document’s event stream and allows to link events to one another (e.g., a tree node to an OTU). References will only be made to previous events that are already known to the application. To reduce the amount of work for application developers, the minimum information is always directly contained in an event object, so that only more complex application models will need to resolve such ID dependencies.

A web interface that generates lists of *JPhyloIO* events from any example file is available at http://r.bioinfweb.info/JPhyloIOEventLister. It allows to fold and unfold the output of subsequences and helps to get started with the way *JPhyloIO* translates phylogenetic data and metadata into events. Besides using custom files, users can also choose from a set of predefined example files in different formats.

### Data adapters for writing documents

**Fig. 5:**
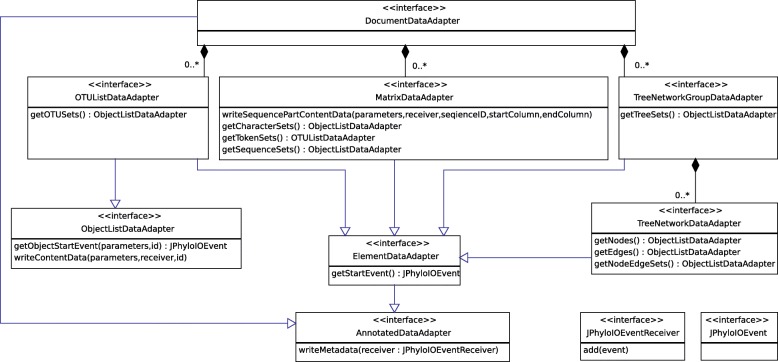
UML diagram showing the data adapter interfaces providing access to the application model for *JPhyloIO* writers. From top to bottom the object relation (indicated by compositions) is shown, while the class hierarchy can be read from bottom to top. Note that not all but only exemplary methods are shown for each interface. The DocumentDataAdapter is the main adapter that provides access to other adapters modelling OTU lists, matrices and phylogenetic trees or networks. Not all application models will provide all these datatypes and therefore not need to implement all types of adapters. The format specific writer classes in *JPhyloIO* can access the data either by event getter methods (e.g., MatrixDataAdapter.getSequenceStartEvent()) with an event ID as its parameter or by writeXXX() methods (e.g., MatrixDataAdapter.writeSequencePart-ContentData()), which write a whole subsequence of the event stream to a special receiver object provided by the application. To simplify the adapter implementation for application developers only frequently used events are provided by getter methods, while the others can directly be written in a sequence by implementing an appropriate writer method. (Getter methods were introduced for cases where random access to events with known IDs is frequently necessary for writers, to avoid requesting a whole sequence, if only one event is needed. Providing some events by getter and some by writer methods in the data adapter model is a compromise between ease of implementation and runtime performance.). Some adapters share common functionality, which is modelled by common superinterfaces, such as AnnotatedDataAdapter or ElementDataAdapter

Format-independent writing of phylogenetic data cannot be implemented in as straightforward a manner as, e.g., *StAX* writing for *XML*, since the required order of the data elements varies between the different target formats, and direct writing of an event stream (as defined by the grammar in Fig. 3) is not possible without having to buffer large amounts of data in some cases. Therefore, we provide adapter interfaces to be implemented by an application. These bridge between the application data model and *JPhyloIO* writers (Fig. 2, Fig. 5), which then can request certain subsequences of the event stream (which correspond to a grammar node in Fig. 3) in the order required by their target format.

Implementing such data adapters may be slightly more effort for application developers than just writing a method that directly creates an event stream from their data model, but it has the advantage of allowing unbuffered access in the required order for all formats. Therefore, writing becomes much more memory efficient, especially for large datasets.

### Generalization over different metadata concepts

A key feature of *JPhyloIO* is that it provides a general way of attaching metadata to any element in a phylogenetic data set, thereby abstracting over different metadata concepts found in the supported formats. In our opinion, the *RDF*-based metadata tags used by *NeXML* [39] represent the most powerful way of modelling metadata and therefore were chosen as the foundation of our general concept. It allows to link external resources and to represent trees of hierarchical metadata that can be attached to any element of a phylogenetic document. Predicates from externally defined ontologies are used to link data and metadata, which ensures both maximal flexibility regarding the type of metadata and unambiguous and machine-readable descriptions of the relationships at the same time. Technically, *RDF* distinguishes between “resource metadata elements” that link external resources or a set of other internal metadata elements (forming a subtree within a metadata tree) and “literal metadata elements” that link concrete values (e.g., strings or integers).

Following this structure, *JPhyloIO* provides a resource- and a literal metadata event class. As shown in Fig. 3, the event grammar allows nesting sequences of metadata events (represented by the grammar node MetaInformation) in all data elements. Metadata can either be represented as a resource- or a literal metadata event. The literal metadata objects may be simple values (e.g., numbers or strings) or complex *XML* data, modelled by a sequence of respective events. As an alternative to processing the *JPhyloIO* event stream directly, our library provides adapter classes between *JPhyloIO* readers and both iterator- and cursor-based *StAX* readers and writers to empower application developers to directly reuse possibly existing code for *StAX*-based reading and writing of respective data without the need for any adaptation to *JPhyloIO*.

### Ways to extend *JPhyloIO*

All readers and writers in *JPhyloIO* implement common interfaces and several abstract implementations of these are available that provide shared functionality, e.g., specific for processing text or *XML* formats. It is therefore simplified for developers to add new readers and writers for additional (custom) formats that integrate seamlessly with the architecture of the library and can directly be used with all *JPhyloIO*-dependent code in other software.

For creating complex *Java* objects from metadata event sequences or to write them back, an interface with a set of default implementations for common types is provided, which can be used for additional custom implementations.

*NEXUS*-related classes are designed to use individual handlers for all *NEXUS* blocks and commands, allowing to easily add support for new or custom *NEXUS* elements in third party modules.

## Results

*JPhyloIO* is an open-source programming library that allows to read and write different phylogenetic file formats using a single event-based interface as described above. It covers taxon- or OTU lists, character matrices or multiple sequence alignments, phylogenetic trees or networks and sets of elements (e.g., character sets). Simple annotations and more complex metadata can be attached to all elements of a document (e.g., trees, tree branches, sequences) and *JPhyloIO* translates these using the available features of each supported format.

Source codes and binary distributions are available under the terms of the *GNU Lesser General Public License 3* from http://bioinfweb.info/JPhyloIO/. This website also provides an extensive documentation, including a detailed *JavaDoc* and a set of example applications.

### Supported formats

As shown in Table 1, *JPhyloIO* supports reading and writing the majority of phylogenetic file formats, including common extensions of these. Additionally, reading of some application-specific alignment and tree formats is possible. The library imposes no restrictions on alphabets used in molecular, morphological and other character matrices, but guarantees that no invalid output for any of the target formats can be written.

Sequence data, including optional comments, can be read from and written to the *FASTA* format, with optional column indices at the beginning of each line being processed correctly. Writing of sequences and optional comments is supported, but generated files will never contain column indices, since these are not widely supported and may cause problems in other software.

The *PHYLIP* format exists in a standard [36] and a relaxed [44] variant, which can both be read in interleaved and non-interleaved forms (the non-interleaved form is written for both). The *PHYLIP* format allows sequence names only up to a certain length (which can be longer in the relaxed variant), resulting in the need to shorten them by *JPhyloIO* writers. In contrast to many other available software tools, this implementation ensures that all written names are unique, even if the full names only differ in characters behind the cut-off position. If sequence names were edited, the application will be informed by a translation object, mapping old to new names.

The *NEXUS* format [38] is a text format consisting of blocks that contain different types of data. Each block consists of a set of *NEXUS* commands. *JPhyloIO* offers readers and writers that support commands of the TAXA block containing taxon lists, the DATA, CHARACTERS and UNALIGNED blocks containing sequence and alignment data, the TREES block containing phylogenetic trees and the SETS block, containing sets of other items. One type of custom NETWORKS blocks containing phylogenetic networks in *eNewick* format is also supported (see below). Sequence data can be in standard or interleaved format with both single character and longer tokens and ambiguous character definitions being supported. Tree nodes can be referenced by the taxon label, the taxon index, or by using a separate translation table. In contrast to other software, *JPhyloIO* supports all three methods both for internal and terminal tree nodes, while translation can be switched off for internal nodes if necessary, e.g., to avoid conflicts between support values and taxon list indices. For the SETS block, character, taxon and tree sets are currently supported. The DISTANCES, ASSUMPTIONS and NOTES blocks are currently not supported. As *NEXUS* files identify all elements by a unique label (instead of distinguishing between labels and IDs as, e.g., in *NeXML*), the respective *JPhyloIO* writer edits labels to be unique if necessary, and reports such changes using the same translation object as the *PHYLIP* writer described above.

In addition to the initial *NEXUS* standard, the TITLE and LINK commands from *Mesquite* [45] that allow linking between blocks (e.g., TAXA blocks can be referenced by CHARACTERS or TREES blocks) and the MIXED sequence datatype extension [46] from *MrBayes* [47] are recognized.

Phylogenetic trees are represented as *Newick* strings [37] in the *TREES* block of a *NEXUS* document or in separate text files containing a set of *Newick* strings separated by semicolons, which are sometimes referred to as *Newick* files and are, e.g., used by *MEGA* [48]. Such *Newick* files are modelled as a separate format in *JPhyloIO* that can be read and written. *Newick* tree definitions (in *Newick* and *NEXUS* files) may contain metadata in hot comments, which can also be read and written. (See below.)

The readers for both *NEXUS* and *Newick* can also read definitions of phylogenetic networks in the *Extended Newick* or *eNewick* format [49] and model its crosslink type (if specified) as metadata.

*NeXML* [39] is a more recent *XML* format that is inspired by *NEXUS* but allows a more advanced way of linking different phylogenetic data elements (e.g., a tree node to an OTU). Additionally, it uses *RDFa* to attach metadata to all elements (trees, alignments, nodes, sequences, …), which provides the basis for the general metadata model used in *JPhyloIO*. (See below.) Readers and writers supporting all features of the format, including its full metadata concept and automated handling of custom sequence tokens, are provided by our library.

*phyloXML* [40] also models complex metadata using a different concept than *NeXML*. It is used to store phylogenetic trees and is fully supported by *JPhyloIO*. Although *phyloXML* uses a hierarchical tree representation, it allows to specify additional clade relation tags to define phylogenetic networks that are used by *JPhyloIO*’s reader and writer.

In addition, readers for some application specific formats are available. For the *MEGA* format [48], a reader provides access to its alignment data and character sets (attached by the LABEL, GENE or DOMAIN commands of the MEGA format). Multiple sequence alignments and attached metadata from *PDE* files produced by the alignment editor *PhyDE* [50] and trees, including their metadata, from *XTG* files used by the phylogenetic tree editor *TreeGraph 2* [51] can be read as well.

### Supported metadata models

Whereas the metadata representation in *NeXML* is by definition identical to *JPhyloIO*‘s *RDF*-based metadata model (as described in “Implementation” above), reading and writing of other formats requires a translation to the respective format-specific model. *FASTA* and *PHYLIP* do not support metadata, so the respective writers ignore provided attachments and log warnings.

*phyloXML* does not use an *RDF*-like concept, but offers a fixed set of metadata elements, stored in special *XML* tags. To access such metadata in *JPhyloIO*, we defined *RDF* predicates for each predefined metadata element for internal use in *JPhyloIO*, to allow identifying the p*hyloXML* tags in our *RDF*-based model. In addition, *phyloXML* offers ways to freely attach metadata by (i) property tags to attach simple annotations (e.g., strings, numeric values or *URI*s) to trees, clades or sequences and (ii) custom *XML* structures added to a whole document, a tree, a clade or some of the predefined annotation tags. *JPhyloIO* makes use of all these features to attach metadata not linked using p*hyloXML*-specific predicates. In combination, this allows to read and write all modelled metadata. Since representing custom hierarchical *RDF* metadata (different from the predefined p*hyloXML* annotation types) is not possible in this format, parts of it will be ignored during writing and respective warnings (similar to *FASTA* and *PHYLIP*) will be logged. Different strategies on how to translate a full *RDFa* annotation tree into *phyloXML* are offered by *JPhyloIO* and can be selected using a writer parameter.

For attaching metadata to nodes and branches in *Newick* strings [37], two extensions that make use of hot comments (comments that contain actual metadata) are supported by *JPhyloIO*. One is “New Hampshire eXtended” or *NHX* [52, 53], a precursor of p*hyloXML* that allows to use a limited set of its predefined annotations, identified by the respective p*hyloXML* predicates in *JPhyloIO*.

The other extension, used by, e.g., *TreeAnnotator* from the *BEAST* package [54] and recent versions of *MrBayes* [47], allows to attach numeric or string values (or arrays of these) to nodes and branches using a free string identifier. These identifiers differ from the *RDF* predicates (used in *NeXML*), since they can have any form and do not need to be *URI*s. To solve this, all meta-events in *JPhyloIO* can carry a string identifier and an *RDF* predicate as alternative descriptions of their relation to their subject. If a string representation is needed for writing and was not provided, the local part of the predicate *CURIE* will be used.

By supporting these two annotation concepts, *JPhyloIO* allows to read and write metadata from and to *Newick* and *NEXUS* files. As in *phyloXML*, hierarchical metadata cannot be written and warnings will be logged.

*JPhyloIO* also reads metadata from the application-specific *XTG* and *PDE* formats. Both formats may contain a fixed set of metadata for some of their elements and according predicates in namespaces for internal use are defined to identify these (the same way as for *phyloXML*). The *XTG* format and *TreeGraph 2* [51] additionally provide the functionality to attach numeric or string annotations to each node or branch of a tree using any string identifier, which are also supported. Basic annotations present in the *MEGA* format (e.g., a description text for a matrix) are read as well.

### Code example

**Fig. 6:**
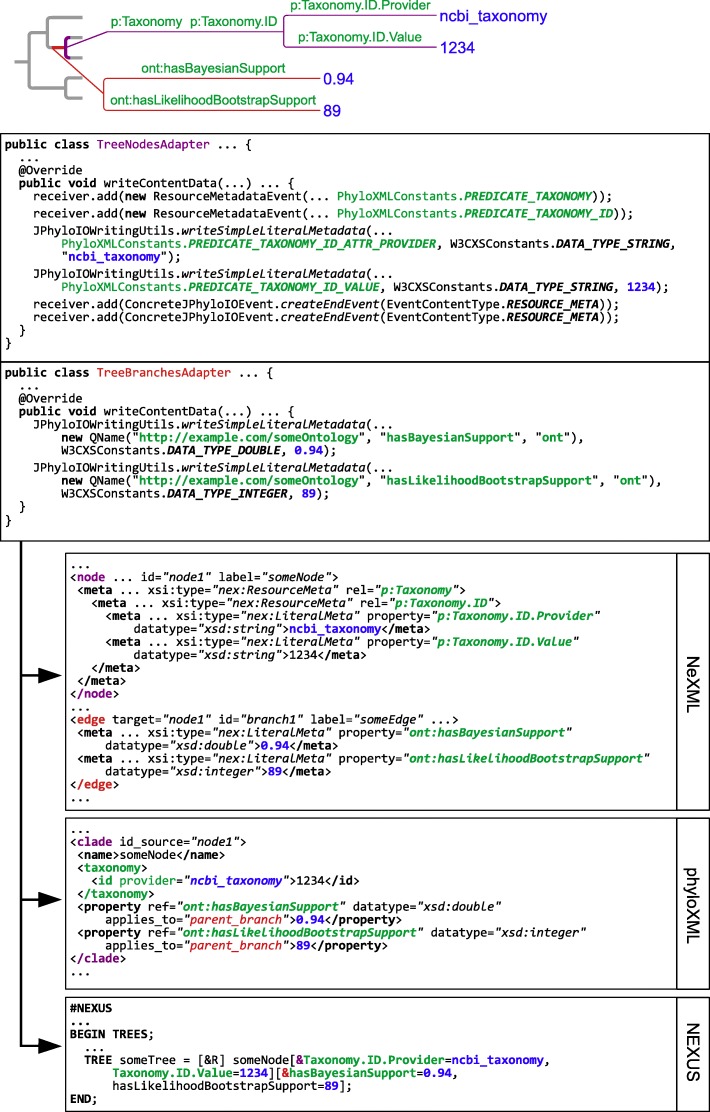
Example code for format-independent metadata writing. The code examples in the two boxes on top show how metadata can be written in *JPhyloIO*. In the lower of the two boxes two support values are attached to a node as literal metadata elements using predicates from a fictional ontology http://example.com/someOntology. The used convenience method writeSimpleLiteralMetadata internally writes a literal metadata start event followed by a respective content and end event as defined by the grammar node LiteralMeta in Fig. 3. The upper box contains an example where literal metadata elements are nested within resource metadata elements. In the concrete example respective predicates for *phyloXML* are used to write an *NCBI* taxonomy ID. The metadata trees attached to the node (in purple) and the branch (in red) are shown on top of the figure. All predicates linking metadata are shown in green, while the actual metadata values are shown in blue. Below the resulting output is shown for three formats. The first box contains *NeXML*, which uses its meta tags to represent the metadata and the linking predicates. The second contains *phyloXML* that uses its specific taxonomy-related elements to represent the node metadata and its property tags to model the branch metadata. (In contrast to *NeXML*, using fully qualified predicates with namespace declarations is not supported.) The box on the bottom contains the *NEXUS* output, where only the metadata from the lowest level is represented using hot comments within the *Newick* string. (Using fully qualified predicates is not possible here either). The full source code and output of this example can be found at http://r.bioinfweb.info/JPhyloIODemoSimpleMetadata. Additional examples for processing data and metadata are also available on the *JPhyloIO* website

Figure 6 provides example code for writing simple and nested metadata attached to one node and one branch of a tree and shows the output in three different formats that result from it. In addition, the documentation on the *JPhyloIO* website contains further code examples that are documented in detail and can be downloaded to test and run them. These are available at http://r.bioinfweb.info/JPIODemo.

### Ways to get involved

Feedback and contributions of the community to the project are made possible by the public bug tracking system on the *JPhyloIO* website, which is also open for feature requests. In addition users can ask questions in the *JPhyloIO ResearchGate* project, using the support e-mail address on the website or via the bioinfweb *Twitter* account. Code contributions are possible within the *JPhyloIO GitHub* repository. (URLs can be found below and on the *JPhyloIO* website.)

## Discussion

Classic formats like *FASTA*, *PHYLIP* or *NEXUS* still play an important role when working with sequences, alignments and phylogenetic trees, mainly because widely used applications often solely rely on these formats (until now). More modern formats with advanced metadata models like *NeXML* or *phyloXML* allow to unambiguously describe and link data and therefore increase its reusability and the reproducibility of underlying studies [13, 32, 55] in a way that would never be possible using only classical format to represent data. *JPhyloIO* generalizes over these different types of file formats while still supporting their individual feature sets. Therefore, it allows bioinformaticians to efficiently develop applications that address both the need for interoperability with widely used software by supporting classical formats and the need for better metadata annotation using modern formats at the same time. This can be achieved by just implementing one interface without having to invest additional resources into each supported format. Developers do not even require detailed knowledge about all formats their applications support, since *JPhyloIO* takes care of which format features to use to optimally represent the content to be written.

Extending existing applications with support for additional, in particular for modern metadata-rich formats, is also simplified by *JPhyloIO*. It integrates well into any existing application data model due to its event-based architecture, which does not impose any requirements on the way data is modelled and stored by an application.

### Comparison with other libraries

Other libraries exist for the *Java* programming language that support reading or writing of alignment or tree formats. *Forester* [56] allows to read and write alignments in *FASTA*, *PHYLIP* and *NEXUS* and phylogenetic trees in *phyloXML*, *NEXUS*, and *NHX.* Phylogenies in the *Tree Of Life Response Format* [57] can be read. The *NeXML* format with its powerful metadata model is not supported and no generalization over the different metadata models exists. The tree readers implement a common interface, but there is no such interface for reading or writing trees and alignments together. As a consequence, *NEXUS* files containing sequence and tree data need to be processed multiple times independently. Unlike *JPhyloIO*, *Forester* enforces its own predefined data model, which can have disadvantages for certain use cases as discussed below. The *NEXUS* TAXA block is only supported when writing trees but not considered for reading trees or for reading and writing alignments, while *NEXUS* sets are not modelled at all.

In its current version 4.2.7, *BioJava* [58] includes only readers and writers for sequence data from the *FASTA* and the *GenBank* format. The *BioJava* legacy version 1.9.2 [59] provides an event/call-back based API through a common interface for some sequence formats, among them the alignment formats *FASTA* and *MSF*, but none of the other formats supported by *JPhyloIO*. Independent readers and writers for *PHYLIP* and *NEXUS* (including support for trees but not for sets) are available, which cannot be accessed through the event-based API. There is no support for *NeXML*, *phyloXML* or complex metadata.

*NeXML* can of course also be read and written using its reference *Java* API [60] implemented together with the publication of the format [39] but this library is not intended to support other formats and abstract over their features.

In other languages, multiple format-specific APIs are available (e.g., [61, 62] and many unpublished ones), some of which also generalize over different formats (e.g., [63–65]).

*BIO::Phylo* [66] is a *Perl* library that supports a number of alignment and tree formats, among them 6 of the 9 formats supported by *JPhyloIO*. Reading and writing is possible through a common interface but a predefined data structure is enforced. Metadata connected using *RDF* predicates is modelled. *phyloXML*-specific predicates are used in a similar way as in *JPhyloIO*, while the set of supported elements is less complete (property and clade_relation tags are not, and legal custom tags are only partly supported). *BIO::Phylo* is able to read (but not write) some types of hot comment tree annotations from *NEXUS*, while *JPhyloIO* supports to read and write a larger set of these.

*NCL* for *C++* [64] supports *FASTA*, *Newick*, *NEXUS* and *PHYLIP.* Plans to support *NeXML* and *phyloXML* were announced in 2010, but have not been implemented as of this writing, and therefore complex metadata is not modeled. Hooks for the application to directly process a whole alignment or a whole tree are provided, but these data elements are much larger than in *JPhyloIO* (where event objects only model, e.g., a short sequence part or a single tree node) and processing of large alignments or trees can be less efficient in *NCL*.

By making use of the *Java Native Interface* (*JNI*) it is possible in principle to access the functionality provided by *JPhyloIO* from nearly all other programming languages. For some languages, special packages are available to make this more convenient, e.g., *Py4J* [67] for *Python* or *rJava* [68] for *R*. It should be noted that making *API* calls this way is usually more intricate than working with *JPhyloIO* in *Java* directly.

Compared to the existing *Java* libraries and even libraries in other languages, *JPhyloIO* supports a large number of formats with a more complete coverage of their feature sets. It does that through a single common interface, while allowing memory efficient event-based processing independent of the application’s data structure. *JPhyloIO*’s generalization over different metadata models, which allows full access to such data from all formats, is currently not offered by any other *Java* library. (As mentioned above, *BIO::Phylo* allows access to a comparable range of formats in *Perl* but is not event-based.)

### Event-based processing versus predefined library data structures

With an event-based architecture as implemented in *JPhyloIO*, application developers can decide for each event how long it should be kept in memory or not. Libraries with predefined data structures load all data from a file into memory at the same time, regardless of the application requirements. This is especially inefficient for use cases that do not need random access to all data (e.g., determining the GC-content of large sequence data sets, searching for certain repeat motives in them or counting the occurrences of a certain node in a large set of trees, e.g., taken as samples from Bayesian phylogenetic inference). Event-based processing reduces the amount of memory needed in such cases from O(n) (linear to the dataset size, e.g., the number of nucleotides) to O(1) (constant, independent of the dataset size), since only the current or a few recent events need to be in memory at once to perform such tasks.

For applications that need random access (e.g., alignment or tree editors), the event-based architecture is still beneficial, because these are often not interested in the whole content of a file (e.g., only in sequence data but not trees) and therefore can directly discard unused events, which they could not do when using library-specific data structures. Even more relevant for complex applications may be the flexibility regarding the data structure. Providing concrete data storage classes with a library, forces applications that need a more advanced or specific model to load the data into instances of library classes first and then copy it into their own specific data structure. This way, the data of at least one file will be in memory twice, which may become a problem for large data sets. Such a problem does not occur with *JPhyloIO*, since event data can directly be stored into any application-specific data structure.

With this in mind, we acknowledge that predefined model implementations may be beneficial for simpler scripts and tools, because developers will not have to deal with implementing their own data structure. Furthermore, creating an application reader for an event stream will usually require a little more effort than simply fetching information from a predefined library data structure, since the reader will need to keep track of the current state, e.g., the current alignment and sequence, in order to process a sequence tokens event. However, this additional effort will not be significant for the majority of more complex applications that benefit from the memory efficiency and flexibility of the data structure and are the main target for *JPhyloIO*. It is also easy to combine *JPhyloIO* with established model standards like the sequence model of *BioJava,* while it still allows to access data not modelled by such third-party libraries.

### Current usage

*JPhyloIO* was developed closely together with *LibrAlign* [69], a *Java* library providing powerful and reusable GUI components for displaying and editing multiple sequence alignments and attached raw- and metadata. To back its GUI components, *LibrAlign* provides a fully implemented data model for sequence and alignment data including ready-to-use reader and writer implementations for *JPhyloIO*.

The *Taxonomic Editor* of the *EDIT platform for Cybertaxonomy* [70] manages taxonomic workflows and their data, while persistently linking character data to preserved individual specimens [30]. *AlignmentComparator* [71] compares alternative multiple sequence alignments of the same dataset. Both make use of *JPhyloIO* and *LibrAlign* for reading and writing alignments and attached metadata. *LibrAlign* and *JPhyloIO* also provide the basis for the alignment editor *PhyDE 2* [72] and they are currently used by our group in the development of tools for the evaluation of automated multiple sequence alignments for phylogenetic purposes and the analysis of microstructural mutational patterns in non-coding DNA.

The tree-related functionality of *JPhyloIO* is the basis in the ongoing metadata model extension in the phylogenetic tree editor *TreeGraph 2* [51]. Versions 2.11.0 and later already use *JPhyloIO* for importing phylogenetic trees and their metadata from *NeXML*. Future versions will adopt the *RDF*-based metadata model into the core data model of the application and simplify meaningful annotation of phylogenetic trees and their nodes and branches using metadata linked with predicates from externally defined ontologies. The generalized metadata model of *JPhyloIO* simplifies importing and exporting metadata for future versions of *TreeGraph 2* significantly.

### Future development

*JPhyloIO* will remain under active development in the future and community contributions are easily possible using *GitHub* and other platforms, as mentioned above. According to the needs of depending software, the library will be adjusted to future changes of the supported formats and be extended to support additional formats. API stability is a key aspect and releases follow the established standard of semantic versioning [73].

As mentioned, the NOTES block of the *NEXUS* format is currently not supported by *JPhyloIO*, although it is used by some applications to store metadata related to data from other *NEXUS* blocks. While the future of metadata representation probably lies in the usage of more advanced formats like *NeXML* or *phyloXML*, having downwards compatibility to the NOTES block might still be beneficial. In contrast the metadata models of the other supported formats (including the hot comments in *NEXUS*), the NOTES block represents a set of metadata elements that usually trail the actual data as a whole and can reference a variety of the previous data elements. In order to model this in *JPhyloIO*, the event grammar would have to be extended to allow metadata events related to a data event at any later position in the stream instead of requiring it to be nested within the respective data events. This would have made developing application readers for the event stream more complicated. The only option to avoid this is to buffer all data until the NOTES block is read, which would destroy the memory efficiency of event-based processing. Based on the requirements of the applications currently based on *JPhyloIO*, we preferred a more concise event grammar and memory efficiency over supporting the *NEXUS* NOTES block. Should the future usage of *JPhyloIO* impose different requirements we would consider to change that strategy and possibly extend the event grammar, ideally in a downwards compatible way.

In addition to the current abstraction over different formats, the abstraction over (future) metadata ontologies relevant for phylogenetics (e.g., possible in *NeXML*) can become a focus. If a critical number of established ontologies will be present, it may be interesting to extend *JPhyloIO* to model equivalent or similar predicates in different ontologies to allow translating between them and to access knowledge in a general way.

## Conclusion

The field of phylogenetics as well as biological sciences as a whole would strongly benefit from a more widespread use of data annotation and respective formats. Unambiguously describing and processing morphological characters and states, documenting voucher information in collections, linking raw data or providing information on the workflow that generated the data are some of many examples where metadata annotation (e.g., using *RDF*) and externally defined ontologies can lead to increased reproducibility of workflows and reusability of data. *JPhyloIO* simplifies writing new and extending existing software that is aimed at achieving this goal by fully supporting metadata-rich formats. Maximum interoperability to older software and downwards compatibility is guaranteed by the parallel support for both advanced and more traditional formats, enabled by the single, format-independent interface of *JPhyloIO*. Developers may support all formats in one step without the need for detailed knowledge on all of them. *JPhyloIO*’s event-based architecture makes integration with any existing application data structure easy and allows very memory-efficient processing even of very large data sets.

### Availability and requirements

**Project name:** *JPhyloIO.*

**Project home page:** http://bioinfweb.info/JPhyloIO/

**GitHub Repository:** https://github.com/bioinfweb/JPhyloIO

**ResearchGate project page:** http://r.bioinfweb.info/RGJPhyloIO

**Operating system(s):** Platform independent.

**Programming language:** *Java.*

**Other requirements:** *Java* Runtime Environment 8 (or higher).

**License:** *GNU Lesser General Public License* Version 3 (*LGPL*).

**Any restrictions to use by non-academics:** The restrictions specified in the *LGPL* apply. (See http://bioinfweb.info/JPhyloIO/License/LGPL.)

## Data Availability

Binary distributions of *JPhyloIO* are available at http://bioinfweb.info/JPhyloIO/Download. Source codes are available at http://bioinfweb.info/JPhyloIO/SourceCode or at the *GitHub* mirror at https://github.com/bioinfweb/JPhyloIO. Unit tests and test data that was used in the development of *JPhyloIO* is available at http://r.bioinfweb.info/JPhyloIOTests.

## Abbreviations

API: Application programming interface
CURIE: Compact Uniform Resource Identifier
DFG: Deutsche Forschungsgemeinschaft (German Research Fundation)
DNA: Deoxyribonucleic acid
FASTA: Fast Adaptive Shrinkage Thresholding Algorithm (This abbreviation is used here for FASTA alignment format.)
GNU: Gnu’s Not Unix (Recursive acronym. Uses a wildebeest (*Connochaetes*) as its icon, which is “Gnu” in German.)
GUI: Graphical User Interface
I/O: Input/Output
ID: Identifier
LGPL: Lesser General Public License
NCBI: National Center for Biotechnology Information
OTU: Operational Taxonomic Unit
OWL: Web Ontology Language (The letter reversal is intended by the authors.)
PhyDE: Phylogenetic Data Editor
RDF: Resource Description Framework
UML: Unified Modeling Language
XML: Extensible Markup Language
XTG: Extensible TreeGraph format
